# Comparative study on the efficacy of different aflibercept regimens in the treatment of macular edema secondary to branch retinal vein occlusion

**DOI:** 10.3389/fmed.2025.1672839

**Published:** 2025-12-05

**Authors:** Xing Du, Yuanyuan Guo, Min Du, Wen Wang, Mingkun Liu, Shanshan Li, Yanjuan Sheng

**Affiliations:** 1 Department of Ophthalmology, Jinan Second People’s Hospital, Jinan, Shandong, China; 2 Department of Hospital Infection Management, Jinan Second People’s Hospital, Jinan, Shandong, China; 3 The Fourth People's Hospital of Jinan, Jinan, Shandong, China; 4 Shandong First Medical University, Jinan, Shandong, China

**Keywords:** aflibercept, branch retinal vein occlusion, macular edema, treat-and-extend, pro re nata, intravitreal injection

## Abstract

**Objectives:**

To compare the efficacy of intravitreal injection of aflibercept between the treat-and-extend (TAE) and pro re nata (PRN, as needed) regimens in the treatment of macular edema secondary to branch retinal vein occlusion, to determine the best regimen in terms of efficacy.

**Methods:**

This was a retrospective case–control study. Totally 62 eyes in 62 patients diagnosed with macular edema secondary to branch retinal vein occlusion (BRVO-ME) from January 2018 to September 2021 were subjected to intravitreal injection of aflibercept according to the different regimens, including the TAE and PRN groups (32 and 30 in the TAE and PRN groups, respectively). Baseline characteristics including age, gender, affected eyes, best corrected visual acuity (BCVA), and central retinal thickness (CRT) were reviewed. The number of injections, number of visits, as well as BCVA and CRT after 12 months of treatment were collected and compared between two groups.

**Results:**

BCVA was 60.7 ± 7.6 and 59.9 ± 6.1 letters at first diagnosis, and 82.1 ± 6.1 and 78.1 ± 5.8 letters after 12 months of treatment in the TAE and PRN groups, respectively. All BCVA was significantly improved from baseline (*p* < 0.01). Based on between-group comparison, BCVA was significantly higher in the TAE group compared with the PRN group (*p* < 0.05). CRT was 483.0 ± 121.5 μm and 478.4 ± 113.0 μm at first diagnosis, and 249.9 ± 30.7 μm and 302.4 ± 41.1 μm after 12 months of treatment in the TAE and PRN groups, respectively. All CRT was substantially reduced from baseline (*p* < 0.01). Based on between-group comparison, CRT was significantly lower in the TAE group compared with the PRN group (*p* < 0.05). The average numbers of visits were 8.6 and 11.5 in the TAE and PRN groups, respectively, indicating a statistically significant difference (*p* < 0.01). The average numbers of injections were 7.6 and 8.1 in the TAE and PRN groups, respectively, with a statistically significant between-group difference (*p* < 0.05).

**Conclusion:**

The TAE regimen is superior to the PRN regimen in terms of efficacy for aflibercept administration in macular edema secondary to branch retinal vein occlusion, with the former regimen also associated with reduced number of injections and visits, as well as decreased burden on patients and physicians.

## 1 Introduction

Branch retinal vein occlusion (BRVO) refers to a complete or partial obstruction at a branch or tributary of the central retinal vein, as a common type of retinal vein occlusion (RVO) (1). BRVO incidence is 1.6% in the Chinese population aged over 40 years, which is 6–7 fold the incidence of central retinal vein occlusion (CRVO) (2). BRVO is associated with visual impairment, which imposes an enormous economic burden on patients and the society (1). Macular edema secondary to BRVO is considered the major cause of visual impairment (1, 3). Current evidence indicates a BRVO-ME incidence of 30% (4). The current management strategy for BRVO is to treat the complications induced by obstruction, i.e., macular edema and neovascularization, rather than the obstruction itself (1, 3). The treatment modalities include anti-vascular endothelial growth factor (anti-VEGF) agents, steroids, laser photocoagulation and operation (3, 5, 6). Intravitreal injection of anti-VEGF agents, such as aflibercept and ranibizumab, has been established as the treatment in BRVO-ME (1, 3). Several treatment regimens for the intravitreal injection of anti-VEGF agents are presently available, including the monthly, PRN and TAE regimens. The TAE and PRN regimens are associated with reduced number of injections and economic burden on patients compared with the monthly regimen. However, the optimal treatment regimen has not been determined for the intravitreal injection of anti-VEGF agents (7), and an increasing number of reports believe that the TAE regimen might be superior and could be applied in clinical practice (8–10). Aflibercept is the first-line drug for the treatment of BRVO-ME (1, 3). Currently, the direct comparative study of the efficacies of aflibercept TAE and PRN regimens in the treatment of BRVO-ME have not been reported. Therefore, the differences in efficacy between these two regimens were compared in this study retrospectively, to determine the optimal regimen.

## 2 Materials and methods

### 2.1 Patient selection

In this retrospective case–control study, 62 eyes in 62 patients diagnosed with BRVO-ME were included in Jinan Second People’s Hospital from January 2018 to September 2021. Among the 32 patients in the TAE group, 17 were males and 15 were females, with a mean age of 63.5 ± 9.4 years. The PRN group consisted of 30 patients, 15 males and 15 females, with a mean age of 63.9 ± 9.2 years (Table 1).

**Table 1:** Observation variables of the TAE group and the PRN group.

Parameter	TAE group (32 eyes)	PRN group (30 eyes)	*P*
Age (years)	63.5 ± 9.4	63.9 ± 9.2	0.88^a^
Gender (Male/Female)	17:15	15:15	0.81^b^
Eye (Left/Right)	16:16	14:16	0.79^b^
Number of visits	8 (1)	11 (1)	<0.01^c^
Number of injections	7 (1)	7 (1)	0.04^c^
BCVA of baseline (letters)	60.7 ± 7.6	59.9 ± 6.1	0.85^d^
BCVA of 12 months (letters)	82.1 ± 6.1	78.1 ± 5.8	0.03^d^
CRT at baseline (μm)	483.0 ± 121.5	478.4 ± 113.0	0.97^d^
CRT at 12 months (μm)	249.9 ± 30.7	302.4 ± 41.1	0.04^d^

a: independent samples *t*-test; b: χ^2^ test; c: Wilcoxon test; d: two-way repeated measures ANOVA.

Inclusion criteria were: (1) age >18 years; (2) diagnosis of BRVO (occlusion of the temporal arcade retinal vein), shown by OCT examination, retinal thickening in the macular area with the foveal center involved; (3) FFA examination shows a non-ischemic BRVO patient. (4) CRT ≥ 300 μm; (5) receive anti-VEGF treatment within 3 weeks after onset. (6) completed treatment with the TAE and PRN regimens for over 12 months, and complete data available.

Exclusion criteria were: (1) previous eye disorders that might affect visual acuity, including diabetic retinopathy and media opacities; (2) as shown by OCT examination, vitrectomy indicated in the presence of epiretinal membrane or vitreomacular traction; (3) intravitreal injection of anti-VEGF agents or glucocorticoids within the three previous months; (4) laser photocoagulation within the three previous months; (5) previous history of shallow anterior chamber or increased intraocular pressure; (6) a medical history of retinopathy that might induce macular edema; (7) a history of thromboembolism.

This study was approved by the Ethics Committee of Jinan Second People’s Hospital (Registration No. 20220610) and complied with the principles of the Declaration of Helsinki. Informed consent was waived by the committee because of the retrospective nature of the study.

### 2.2 Methods

#### 2.2.1 Ophthalmic examinations

All patients were subjected to complete ophthalmic examinations. Best corrected visual acuity (BCVA) was determined as described by the Early Treatment Diabetic Retinopathy Study (ETDRS). Retinal thickness under the fovea was measured with an optical coherence tomography (OCT) instrument (Cirrus OCT from Zeiss, Germany).

#### 2.2.2 Operation

Intravitreal injection was performed by experienced ophthalmologist in an operating room with a sterile laminar flow field. Apply three doses of Oxybuprocaine for superficial esthesia before injection. Routine eye disinfection was carried out, followed by conjunctival sac flushing with the povidone-iodine ophthalmic solution. Aflibercept (Eylea; Bayer Healthcare, Leverkusen, Germany) at 2 mg was injected into the vitreous cavity with a needle. The phakic eye was injected 4 mm behind the limbus, and the intraocular lens eye was 3.5 mm. The anterior index test was then carried out to confirm central retinal artery perfusion. Tobramycin dexamethasone ointment (TobraDex, Alcon) was applied to the conjunctival sac, and the affected eyes were covered with sterile gauze.

#### 2.2.3 Treatments

For the TAE regimen, patients with confirmed diagnosis of BRVO-ME received intravitreal injection of aflibercept at 2 mg at each study visit. All patients received three loading doses at 4-week intervals. Following the loading period, the interval between study visits could be extended by 2 weeks at each visit, to a maximum of 16 weeks, in case of no clinical activity (see below for definition). If clinical activity was present at the end of the loading period, the interval between study visits was kept at 4 weeks until no detected clinical activity, and an attempt to extend the interval was made. If clinical activity recurred during the attempt to extend the interval between study visits, the interval was reduced by 2 weeks. The above procedure was repeated until no detectable clinical activity. The interval between study visits that followed was defined as the maximum interval prior to relapse.

For the PRN regimen, patients with confirmed diagnosis of BRVO-ME received three loading doses at 4-week intervals. Then, during follow-up visits, intravitreal injection of aflibercept was performed based on examination at each visit. Intravitreal injection of aflibercept was performed in case of clinical activity; otherwise, another study visit was scheduled for the following month.

Clinical activity was defined as any of the following: (1) ME on OCT, classified as the presence of intraretinal fluid (IRF) or subretinal fluid (SRF), or CRT increasing by 50 μm or more from the previous visit; (2) reduction in BCVA by 5 or more ETDRS letters from the previous visit.

### 2.3 Statistical analyses

Data were statistically analyzed with SPSS 23.0. *p* < 0.05 was considered statistically significant. Normality distributed data tested by the Shapiro–Wilk test were presented as mean ± standard deviation. BCVA, CRT, and age in both groups were normally distributed, and independent samples *t*-tests were used to compare these data between groups. Inter-group comparison of BCVA and CRT before and after treatment were compared by two-way repeated measures ANOVA. The numbers of injections and visits did not conform to normal distribution, and were presented by Median (IQR) and compared by the Wilcoxon test. Categorical variables, including gender and affected eyes, were presented as frequency, and compared by the Chi test method.

## 3 Results

### 3.1 Changes in BCVA

**Figure 1:**
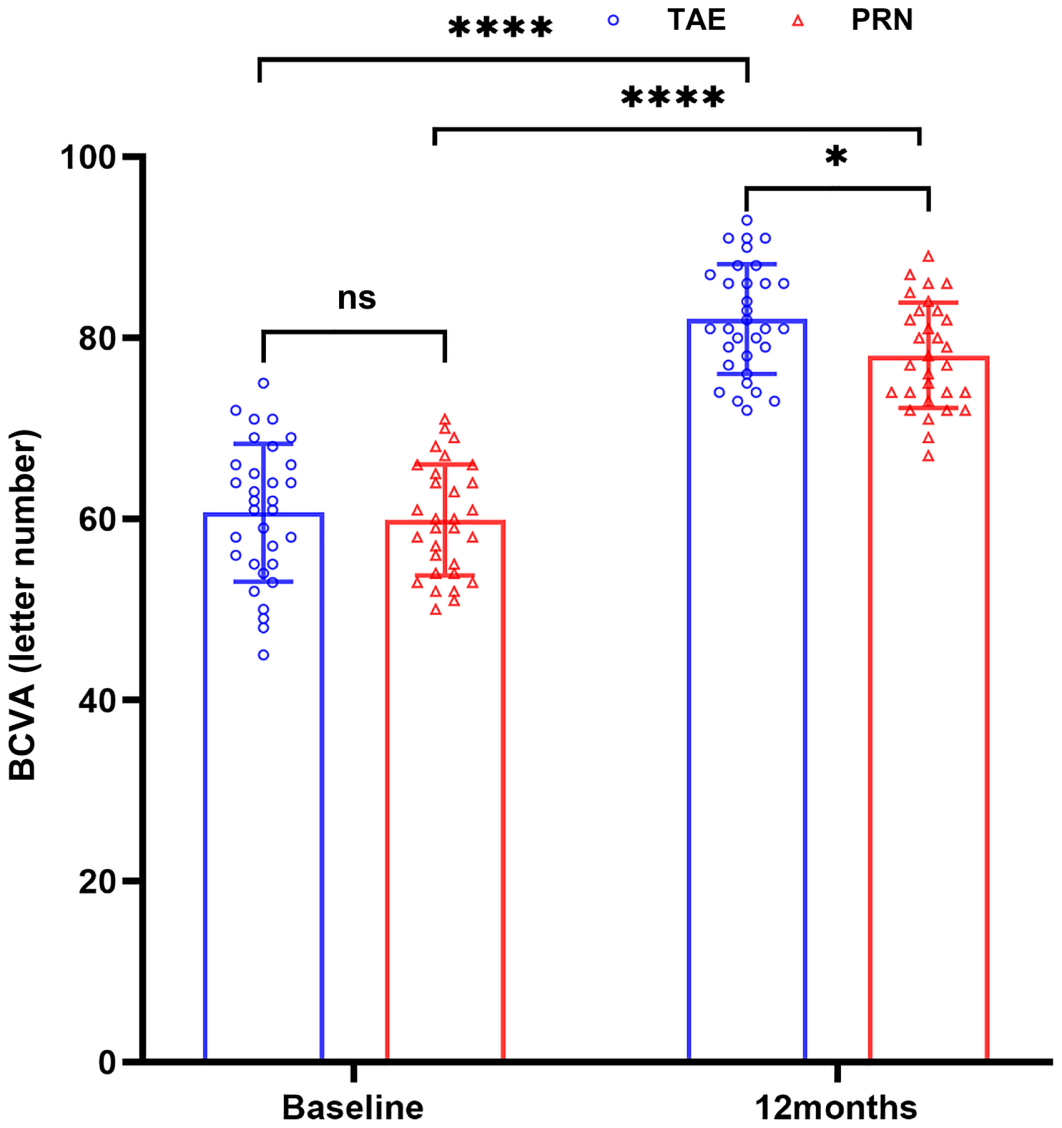
BCVA changes in the TAE and PRN groups after treatment. * Means *p* < 0.05, **** means *p* < 0.0001, ns means no significance. The blue circle represents the BCVA data of 32 patients in the TAE group, and the red triangle represents the BCVA data of 30 patients in the PRN group.

In the TAE group, BCVA was 60.7 ± 7.6 letters at first diagnosis and 82.1 ± 6.1 letters after 12 months of treatment, with an average increment of 21.4 letters, which was improved significantly from baseline (*p* < 0.01). In the PRN group, BCVA was 59.9 ± 6.1 letters at first diagnosis and 78.1 ± 5.8 letters after 12 months of treatment, with an average increment of 18.2 letters, which was improved significantly from baseline (*p* < 0.01). Based on between-group comparison after treatment, BCVA was significantly greater in the TAE group compared with the PRN group (*p* < 0.05). Details are shown in Table 1 and Figure 1.

### 3.2 Changes in CRT

**Figure 2:**
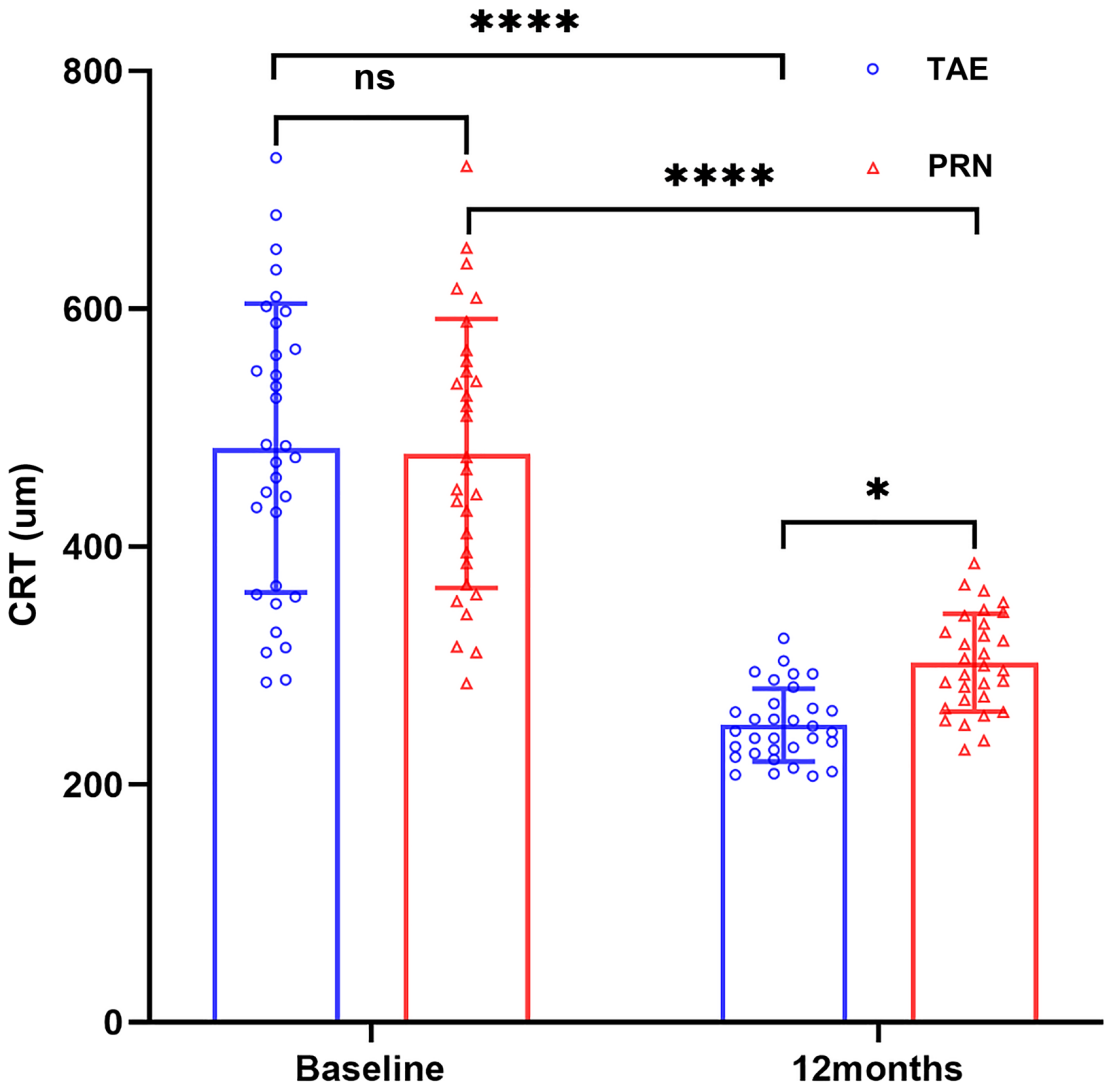
CRT changes in the TAE and PRN groups after treatment. * Means *p* < 0.05, **** means *p* < 0.0001, ns means no significance. The blue circle represents the CRT data of 32 patients in the TAE group, and the red triangle represents the CRT data of 30 patients in the PRN group.

In the TAE group, CRT was 483.0 ± 121.5 μm at first diagnosis, and 249.9 ± 30.7 μm after 12 months of treatment, with an average reduction of 233.1 μm, which indicated a significant decrease from baseline (*p* < 0.01). In the PRN group, CRT was 478.4 ± 113.0 μm at first diagnosis, and 302.4 ± 41.1 μm after 12 months of treatment, with an average reduction of 176 μm, which also indicated a significant decrease (*p* < 0.01). In between-group comparison, CRT was significantly lower in the TAE group compared with the PRN group (*p* < 0.05). Details are shown in Table 1 and Figure 2.

### 3.3 Numbers of injections and visits

After 12 months of treatment, the average numbers of visits were 8.6 and 11.5 in the TAE and PRN groups, respectively. The number of visits was reduced by 2.9 in the TAE group compared with the PRN group, indicating a significant between-group difference (*p* < 0.01). The average numbers of injections were 7.6 and 8.1 in the TAE and PRN groups, respectively. The number of injections was reduced by 0.5 in the TAE group compared with the PRN group, indicating a significant between-group difference (*p* = 0.035).

### 3.4 Adverse reactions

No severe ocular adverse reactions, including secondary glaucoma, vitreous hemorrhage, retinal detachment, endophthalmitis, uveitis and ocular toxicity, were detected in this study. In addition, no systemic complications such as cardiovascular and cerebrovascular diseases were observed.

## 4 Discussion

BRVO is associated with decreased venous outflow within the retinal circulation, thereby increasing retinal capillary pressure and permeability and resulting in retinal leakage (11). Retinal nonperfusion resulting from occlusion induces different degrees of retinal ischemia, depending on the extent of thrombus formation and occlusion site (11). Retinal ischemia is associated with the synthesis of VEGF (12), which enhances retinal microvascular permeability and subsequently aggravates macular edema and neovascularization (11, 12). Moreover, macular edema is associated with visual impairment (11). As shown by Noma et al. (13), VEGF is upregulated in the aqueous humor and vitreous body of RVO patients, and there is a positive correlation between VEGF levels and the degree of macular edema. Aflibercept, a recombinant fusion protein consisting of the binding domains of VEGFR-1 and VEGFR-2, as well as the Fc portion of IgG1, exhibits high affinity to VEGF-A, VEGF-B and PIGF, preventing the binding of these factors and activating their cognate receptors (14). Intravitreal injection of aflibercept for the treatment of BRVO-ME is well established, as well as it pharmacodynamic effects (15). As shown above, BCVA and macular edema were significantly improved after treatment with aflibercept for 12 months compared to baseline, which was consistent with the studies by Roderick et al. (7, 8). After treatment for 12 months, BCVA was increased by 21.4 letters and 18.2 letters on average in the TAE and PRN groups, respectively, indicating that intravitreal injection of aflibercept significantly improves visual acuity in patients with BRVO. Meanwhile, after treatment for 12 months, CRT was reduced by 233.1 μm and 176 μm on average in the TAE and PRN groups, respectively, indicating that intravitreal injection of aflibercept significantly improves macular edema and restores normal retinal anatomy. The therapeutic effects of both treatment regimens were directly compared, and the TAE group exhibited more favorable profiles in both BCVA and CRT in comparison with the PRN group, with statistically significant differences. These data suggest that the TAE regimen of aflibercept provides more pronounced improvements in visual function and anatomy versus the PRN regimen. As a passive treatment regimen, the PRN regimen was administered only when patients experienced recurrence of macular edema or vision loss, which was considered a possible reason behind the poorer efficacy of the PRN regimen compared with the TAE regimen. Additionally, the visual potential and ultimate visual outcome of aflibercept therapy were also restricted by recurrent macular edema. As shown in the COPERNICUS and GALILEO studies, visual and anatomical improvements achieved by the monthly regimen could not be maintained by the PRN regimen (16, 17), in agreement with this study. As an individualized active treatment regimen, the TAE regimen was started from monthly injections, and the treatment interval was gradually extended until an optimal interval for maintaining efficacy was determined. In case of clinical activity, the interval between study visits shall be reduced appropriately based on treatment requirements. In the TAE regimen, aflibercept efficacy and the individual medical needs of patients were aligned as much as possible to reduce the number of injections into eyes with low clinical activity and to allow eyes with high clinical activity to receive more frequent injections.

Additionally, the numbers of injections and visits were lower in the TAE regimen compared with the PRN regimen, especially the number of visits. Therefore, the TAE regimen might impose lower medical burden on both patients and physicians versus the PRN regimen. All patients in this study received three loading doses at 4-week intervals. If the loading period was further shortened, the numbers of visits and injections would be further reduced, which warrants further validation by clinical studies.

In this study, no severe ocular adverse reactions were reported, including secondary glaucoma, vitreous hemorrhage, retinal detachment, endophthalmitis, uveitis and ocular toxicity, as well as systemic complications. These results were consistent with Park et al. (18, 19).

The strength of this study was its control- and single-center nature, and the treatment outcomes of different aflibercept regimens were directly compared. However, there were some limitations. As a retrospective study, only patients with complete data were analyzed, and bias was unavoidable. Additionally, due to the small sample size, short follow-up duration, and an absence of patients with CRVO, these results should be interpreted with caution. In the future, large multi-center prospective randomized controlled studies are warranted to provide more evidence.

In this retrospective study, the TAE regimen for aflibercept is superior to the PRN regimen in terms of efficacy in the treatment of macular edema secondary to branch retinal vein occlusion. This regimen is also associated with reduced numbers of injections and visits, as well as decreased burden on healthcare professionals and patients.

## 5 Contribution to the field

Branch retinal vein occlusion (BRVO) is a subtype of retinal vein occlusion that occurs when there is a partial or complete blockage in a branch or tributary of the central retinal vein. Visual impairment associated with BRVO imposes a significant economic burden on patients and society, with macular edema (BRVO-ME) being the leading cause of this impairment, especially in developing countries (20, 21). The first-line treatment for BRVO-ME is intravitreal injection of anti-VEGF agents, such as aflibercept, which has been shown to effectively improve both visual acuity and anatomic structure. Currently, different treatment regimens exist for intravitreal injection of anti-VEGF agents, such as the monthly, PRN and TAE regimens. In this retrospective case-control study, we compared the effectiveness of intravitreal injection of aflibercept between TAE and PRN regimens in the treatment of BRVO-ME. Due to the shortage of medical resources in developing countries (20, 21), it is particularly important to develop better treatment schemes with less economic burden. Therefore, our research can provide clinical data support. Our results suggested that in terms of efficacy for aflibercept administration in BRVO-ME, the TAE regimen has been shown to be superior to PRN regimen. Additionally, the TAE regimen is associated with a reduced number of injections and visits, resulting in decreased burden on both patients and physicians.

## Data Availability

The original contributions presented in the study are included in the article/supplementary material, further inquiries can be directed to the corresponding author.
